# Mendelian Randomization in Stroke: A Powerful Approach to Causal Inference and Drug Target Validation

**DOI:** 10.3389/fgene.2021.683082

**Published:** 2021-08-12

**Authors:** Julián N. Acosta, Natalia Szejko, Guido J. Falcone

**Affiliations:** ^1^Division of Neurocritical Care and Emergency Neurology, Department of Neurology, Yale School of Medicine, New Haven, CT, United States; ^2^Department of Neurology, Medical University of Warsaw, Warsaw, Poland; ^3^Department of Bioethics, Medical University of Warsaw, Warsaw, Poland

**Keywords:** stroke, genetics, Mendelian randomization, polygenic risk scores, drug target validation

## Abstract

Stroke is a leading cause of death and disability worldwide. However, our understanding of its underlying biology and the number of available treatment options remain limited. Mendelian randomization (MR) offers a powerful approach to identify novel biological pathways and therapeutic targets for this disease. Around ~100 MR studies have been conducted so far to explore, confirm, and quantify causal relationships between several exposures and risk of stroke. In this review, we summarize the current evidence arising from these studies, including those investigating ischemic stroke, hemorrhagic stroke, or both. We highlight the different types of exposures that are currently under study, ranging from well-known cardiovascular risk factors to less established inflammation-related mechanisms. Finally, we provide an overview of future avenues of research and novel approaches, including drug target validation MR, which is poised to have a substantial impact on drug development and drug repurposing.

## Introduction

Stroke is a leading cause of mortality and disability worldwide. While the overall incidence of stroke is decreasing, the global burden of this disease remains high because the absolute number of disability-adjusted life years lost due to stroke is increasing as the population grows and ages (Johnson et al., 2019). Thus, there is an urgent need for novel preventive, therapeutic, and rehabilitation strategies. Among these three, new acute treatments are particularly needed, as the few alternatives available thus far include thrombolytic therapy and mechanical thrombectomy for ischemic stroke (Powers et al., 2019), targeted blood pressure reduction for intracerebral hemorrhage (ICH; Hemphill et al., 2015), and early securing of the aneurysm in subarachnoid hemorrhage (Connolly et al., 2012).

In this setting, the field of stroke research can greatly benefit from the tools that population genetics has to offer. Mendelian randomization (MR) is a statistical method aimed at determining and quantifying causal relationships between genetically determined exposures and outcomes of interest (Davies et al., 2018). Importantly, in contrast to randomized clinical trials (the most frequently used tool to evaluate causality), MR can be performed using already available open-access data from different sources, allowing the evaluation of larger numbers of possible mechanisms and accelerating the speed of the translational cycle.

In this review, we introduced the concept of MR and provide an overview of the existing MR studies focused on stroke. To identify these studies, we conducted a systematic search of the medical literature using PubMed and the keywords “Mendelian randomization stroke,” “Mendelian randomization intracerebral hemorrhage,” and “Mendelian randomization subarachnoid hemorrhage” in the MEDLINE database. We also explored some analytical possibilities beyond classical MR, including the use of multiomics data and drug target validation MR.

## Mendelian Randomization: A Primer

**Figure 1:**
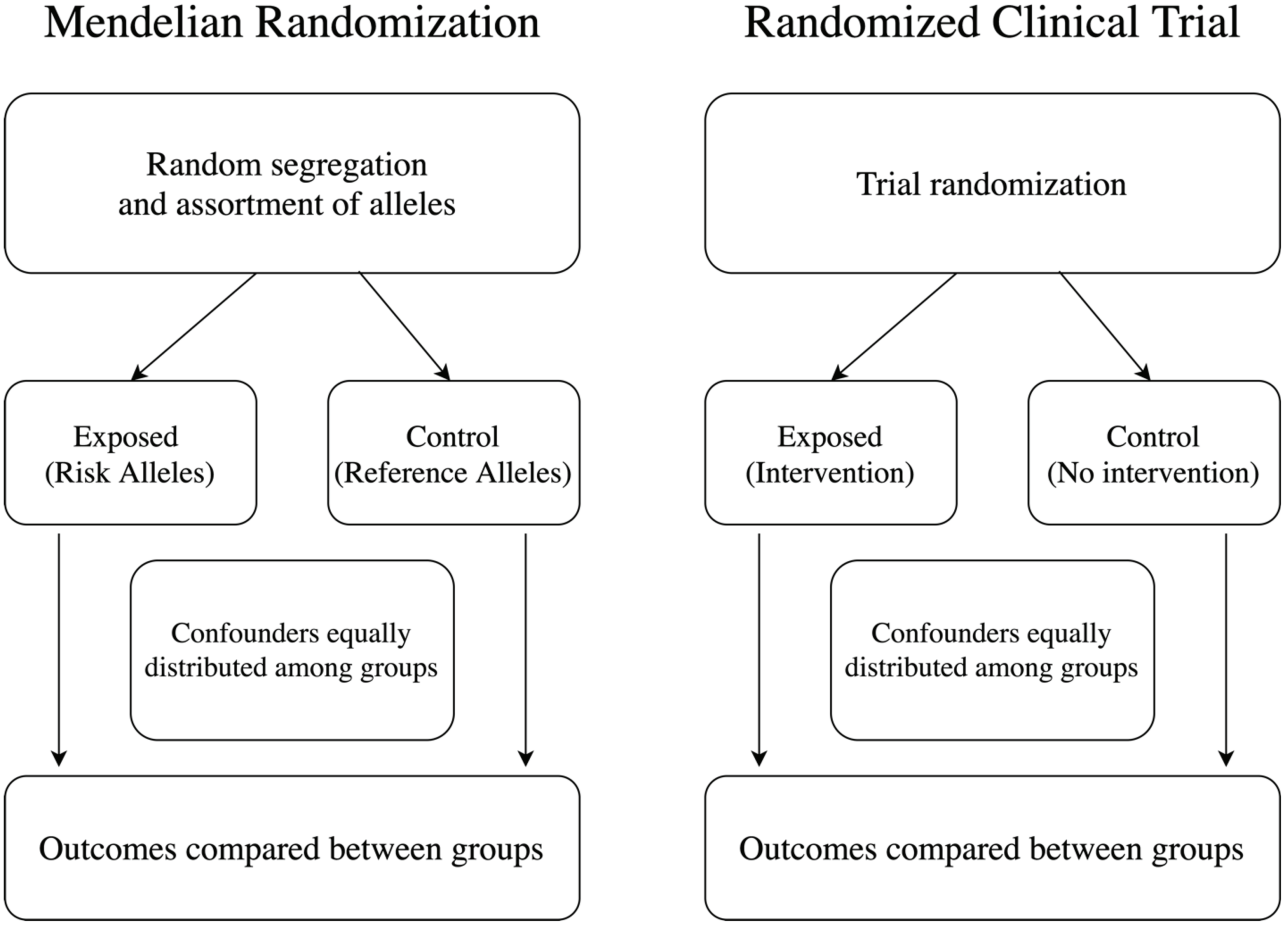
Mendelian randomization (MR) as instrument variable analysis. MR is a type of instrumental variable analysis, similar to randomized clinical trials. Exposed individuals in MR are those carrying risk alleles for determined genetic variants known to associate with an exposure of interest.

MR constitutes a special case of instrumental variable analysis, a widely used analytical framework for causal inference. When the MR assumptions are met, it is possible to identify and quantify causal relationships between exposures and outcomes of interest (Figure 1). MR is best explained using an example. For this section, we will use the link between cholesterol levels and systolic blood pressure as our starting point. In randomized clinical trials, investigators randomly assign study participants to an intervention or placebo to study causal relationships between the intervention and the outcome of interest. In our example, researchers would randomly assign participants to receive a cholesterol-lowering drug or placebo and then measure systolic blood pressure levels in both groups. In MR analysis, we would use genetic variants strongly associated with the exposure of interest as the instrument. In this case, we would choose genetic variants strongly associated with cholesterol levels. As these variants are randomly segregated before birth, one could then separate groups according to the number of risk alleles, and the resulting analyses would not be confounded by environmental exposures happening after birth. Thus, we can measure systolic blood pressure levels in those groups and implement the necessary comparisons.

**Figure 2:**
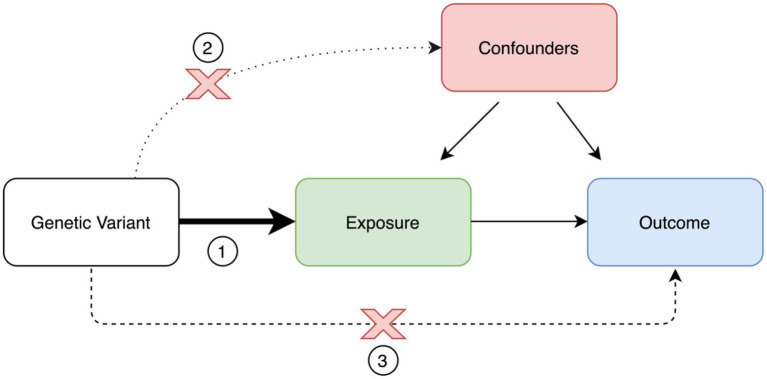
Mendelian randomization paradigm and assumptions. Mendelian randomization assumptions: (1) valid instrument, (2) no association with confounders, and (3) no horizontal pleiotropy.

The implementation of MR analyses requires important assumptions (Figure 2). First, the genetic variants used as instruments must be strongly associated with the exposure or risk factor of interest. This is generally easily accomplished, since these variants are often derived from large-scale genome-wide association studies (GWAS) of the exposure of interest. Second, there should be no confounders affecting the association between genetic variants and the outcome of interest. This assumption is not trivial, and, at the moment, there is no method to definitively confirm this assumption. Although not always performed, a practical way to tackle this problem is to test the genetic variants used as instruments against an array of different other potential covariates that could lead to bias. Third, the genetic variants used as instruments affect the outcome of interest only *via* the exposure of interest, with no other alternative pathways coming into play. These alternative pathways are part of what is called horizontal pleiotropy. There are several statistical methods to test and correct for horizontal pleiotropy, including the Mendelian randomization–Egger (MR–Egger) intercept, MR Pleiotropy RESidual Sum and Outlier (MR-PRESSO), and Causal Analysis Using Summary Effect Estimates (CAUSE; Bowden et al., 2015; Verbanck et al., 2018; Morrison et al., 2020).

Crucial to MR studies is the online availability of open-access resources, including tools to rapidly test hypotheses such as MR-Base (Hemani et al., 2018); large biobanks such as the UK Biobank (Bycroft et al., 2018), the China Kadoorie Biobank (CKB; Chen et al., 2011), Biobank Japan (Nagai et al., 2017), and the All of Us Research Program (Denny et al., 2019); and repositories of summary statistics from large GWAS like the GWAS catalog (Buniello et al., 2019), the GWAS atlas (Tian et al., 2020), and specifically for stroke, the Cerebrovascular Disease Knowledge Portal (Crawford et al., 2018). These and other helpful resources are summarized in Table 1.

**Table 1:** Online resources available for Mendelian Randomization studies.

References	Name	Website	Description
Crawford et al., 2018	Cerebrovascular Disease Knowledge Portal	http://www.cerebrovascularportal.org/	A platform that allows for searching, visualizing, and analyzing variations related to cerebrovascular disease.
Buniello et al., 2019	GWAS Catalog	https://www.ebi.ac.uk/gwas/	Catalog of GWAS results
Tian et al., 2020	GWAS atlas	https://atlas.ctglab.nl/	Catalog of GWAS results
Lambert et al., 2019	PGS Catalog	http://www.pgscatalog.org/	Catalog of Polygenic Risk Scores
Hemani et al., 2018	MR-base	https://www.mrbase.org/	A database and analytical platform for Mendelian Randomization
Bycroft et al., 2018	UK Biobank	https://www.ukbiobank.ac.uk/	Observational study, enrolling >500,000 participants, open-access
Gaziano et al., 2016	Million Veteran Program	https://www.research.va.gov/mvp/	Observational study, enrolling 1 million participants, restricted access
Denny et al., 2019	All of Us	https://allofus.nih.gov/	Observational study, aiming to enroll 1 million participants, open-access
Chen et al., 2011	China Kadoorie Biobank	https://www.ckbiobank.org/	Observational study, 500,000 participants, restricted access
Nagai et al., 2017	Biobank Japan	https://biobankjp.org/english/index.html	Observational study, ~200,000 participants, restricted access
Wong et al., 2017	dbGAP	https://www.ncbi.nlm.nih.gov/gap/	Repository, open-access
Lappalainen et al., 2015	EGA	https://ega-archive.org/	Repository, open-access

## Traditional Cardiovascular Risk Factors

Cardiovascular risk factors represent the most important determinants of risk of stroke. While several observational studies have shown associations between traditional vascular risk factors and risk of different types of stroke, deriving causality from observational data is problematic due to the possibility of bias. In the last 5 years, several MR studies have been conducted to explore, confirm, and quantify causality behind these already described associations (Table 2).

**Table 2:** Summary of MR studies looking at traditional risk factors for stroke.

References	Exposure	Outcomes	Findings
Georgakis et al., 2020b	Blood pressure levels	Any stroke, ischemic stroke, LAS, CES, and SVS	Ten mmHg increase in SBP was associated with approximately 40% increase in the risk of any stroke or ischemic stroke. It was also associated with the risk of stroke subtypes, except for lobar ICH. Similar findings were presented for DBP. Decreases in SBP through calcium channel blockers but not through beta-blockers was associated with decrease in ischemic stroke risk.
Georgakis et al., 2020a	PP	Ischemic stroke and subtypes	Pulse pressure was independently associated with stroke risk in participants older than 55 years.
Hindy et al., 2018	LDL-C	Ischemic stroke and subtypes	Higher LDL-C was associated with higher risk of ischemic stroke and LAS.
Allara et al., 2019	Lipid fractions levels	CVD outcomes	Higher LDL-C was associated with higher risk of ischemic stroke. Higher HDL-C levels were associated with lower risk of ICH. Higher triglycerides were associated with lower risk of ICH.
Valdes-Marquez et al., 2019	LDL-C	CHD, Ischemic stroke and its subtypes	LDL-C levels were not associated with the risk of ischemic stroke or its subtypes.
Georgakis et al., 2020c	Lipid fractions levels	SVS, WMH volume, and ICH	Higher HDL-C levels were associated with lower risk of SVS and lower WMH volumes. Higher HDL-C levels were associated with higher risk of ICH.
Sun et al., 2019	Lipid fractions levels	Ischemic stroke and ICH	Higher LDL-C levels were associated with higher risk of ischemic stroke and lower risk of ICH.
Falcone et al., 2020	Lipid fractions levels	ICH	Higher LDL-C levels were associated with lower risk of ICH.
Pan et al., 2019	Lp(a)	Ischemic stroke, its subtypes, AD	Lp(a) levels were associated with higher risk of LAS, but lower risk of SVS and Alzheimer’s disease.
Larsson et al., 2020b	Lp(a)	Ischemic stroke, AD, parental AD or dementia	Lp(a) levels were associated with the risk of ischemic stroke. There is an inverse relationship between Lp(a) and Parental AD or dementia.
Larsson et al., 2017	T2DM	Ischemic stroke and its subtypes	T2DM was associated with the risk of ischemic stroke, specially LAS.
Gan et al., 2019	T2DM	Ischemic stroke	T2DM was associated with ischemic stroke.
Liu et al., 2018	T2DM	Small vessel disease phenotypes	T2DM was associated with MRI-confirmed lacunar stroke.
Chen et al., 2020	Insulin resistance	Ischemic stroke and subtypes	Insulin resistance was associated with ischemic stroke, particularly SVS.
Yeung et al., 2018	HbA1c	Ischemic stroke	No association.
Georgakis et al., 2021	Type 2 diabetes, HbA1c, insulin resistance, and β-cell dysfunction	Ischemic stroke, ischemic stroke subtypes, intracerebral hemorrhage, related phenotypes	T2DM and higher HbA1c levels were associated with higher risk of ischemic stroke, especially LAS and SVS. Insulin resistance and β-cell dysfunction show similar associations, with the latter also associated with intracerebral hemorrhage. T2DM was also associated with lower white matter integrity (fractional anisotropy). T2DM, HbA1c, and β-cell dysfunction were associated with lower grey matter volume and total brain volume.
Qian et al., 2019	Smoking	Ischemic stroke	Smoking was associated with any ischemic stroke and LAS.
Larsson et al., 2019a	Smoking initiation	Ischemic stroke, subtypes, and ICH	Smoking initiation was associated with ischemic stroke, LAS, and SVS, but not with CES or ICH.
Larsson et al., 2020c	Smoking	14 CVDs	Smoking was associated with a broad range of CVDs including coronary artery disease, heart failure, abdominal aortic aneurysm, ischemic stroke, transient ischemic attack, peripheral arterial disease, and arterial hypertension.
Acosta et al., 2021	Smoking initiation	SAH	Smoking initiation was associated with the risk of nontraumatic SAH.
Dale et al., 2017	General adiposity and central adiposity	Stroke	Central adiposity but not general adiposity was associated with stroke risk.
Marini et al., 2020	BMI and WHR	Ischemic stroke, subtypes, ICH, WMH volume	Higher WHR but not higher BMI was associated with all-cause ischemic stroke, LAS, SVS, non-lobar ICH and WMH volume.
Zhuang et al., 2020	Physical activity	Stroke	No association.
Hou et al., 2020	Atrial fibrillation	CES	Bidirectional association between atrial fibrillation and CES.

*CVD, cardiovascular disease; ICH, intracerebral hemorrhage; LAS, large artery stroke; SVS, small vessel stroke; CES, cardioembolic stroke; WMH, white matter hyperintensity; SAH, subarachnoid hemorrhage; PP, pulse pressure; HDL-C, high-density lipoproteins cholesterol; LDL-C, low-density lipoprotein cholesterol; Lp(a), lipoprotein(a); T2DM, type 2 diabetes mellitus; HbA1c, glycosylated hemoglobin; BMI, body mass index; and WHR, waist-to-hip ratio*.

### Blood Pressure

Robust existing evidence indicates that high blood pressure is one of the main risk factors for stroke. However, several questions remain, including how early and sustained changes in blood pressure affect outcomes later in life and whether specific pharmacological interventions provide additional benefits over others. Georgakis et al. (2020b) utilized MR analyses to assess the relationship between genetically proxied blood pressure levels and the risk of any stroke as well as its subtypes. They demonstrated that blood pressure levels were causally associated with the risk of any stroke and most subtypes, with the exception of lobar ICH. The effect of blood pressure appeared stronger in large artery stroke (LAS) and small vessel stroke (SVS). Additionally, the authors explored genetic proxies for two different antihypertensive drugs classes, calcium channel blockers and beta-blockers, finding that a 10 mmHg reduction in systolic blood pressure using genetic proxies for calcium channel blockers was protective for any stroke and its subtypes, especially for SVS (40% reduction). Furthermore, the same reduction in blood pressure through calcium channel blockers variants was also associated with lower white matter hyperintensity (WMH) volumes, a well-established neuroimaging biomarker for cerebral small vessel disease. A study from the same group also evaluated whether blood pressure pulsatility (i.e., pulse pressure) affects stroke risk independently of the mean arterial pressure. Using multivariable MR within the UK Biobank, they found that pulse pressure was independently associated with ischemic stroke risk (but not ICH) in participants older than 55 years, being particularly important for LAS (Georgakis et al., 2020a). Beyond ischemic stroke, MR analyses have confirmed observational evidence that indicate that high blood pressure is associated with higher risks of intracranial aneurysms and subarachnoid hemorrhage (Bakker et al., 2020).

### Lipids

The role of lipids in cardiovascular disease (CVD) has been extensively studied. However, the effect of circulating lipid levels on stroke appear to be heterogeneous and depend on the specific subtypes being evaluated. Hindy et al. (2018) investigated the effect of blood lipids on the risk of ischemic stroke and its subtypes using summary statistics from the GWAS completed by the Stroke Genetics Network (SiGN), which included 17,000 ischemic stroke cases and more than 32,000 controls of European ancestry. They found that low-density lipoprotein cholesterol (LDL-C) levels were associated with risk of ischemic stroke, especially LAS. HDL-C levels were inversely associated with the risk of SVS. There were no consistent associations for triglycerides. More recently, Allara et al. (2019) conducted a two-sample MR study to identify relationships between lipid levels and the risk of several cardiovascular outcomes, including stroke. In their study, higher HDL-C levels were associated with lower risk of ICH, while higher LDL-C levels were associated with higher risk of ischemic cerebrovascular disease. Interestingly, they also found an inverse association between triglycerides and the risk of ICH. A rigorous study performed by Valdes-Marquez et al. (2019) investigated the effect of LDL-C on the risk of ischemic stroke and its subtypes and did not find any significant associations with these conditions. Georgakis et al. (2020c) also looked at the association between lipid fractions and small vessel disease, a broad phenotype that includes SVS, white matter disease, and ICH. They found that higher HDL-C was associated with lower risk of SVS and lower WMH volume. Interestingly, they also found a relationship between higher HDL-C and higher risk of ICH. Along this lines, Sun et al. (2019) found a strong positive association between LDL-C and ischemic stroke and a strong inverse association with ICH in participants of the China Kadoorie Biobank. This inverse association between genetically determined LDL-C levels and the risk of ICH was confirmed in cohorts of European descent by Falcone et al. (2020). The latter two studies confirmed previous observational results that suggested that very low LDL-C levels increase the risk of ICH (Wang et al., 2013; Saliba et al., 2018; Ma et al., 2019), pointing to novel pathways in ICH.

Another study recently explored association pertains to lipoprotein(a) [Lp(a)] levels and ischemic stroke. Using summary statistics from MEGASTROKE and the International Genomics of Alzheimer’s Project (IGAP), Pan et al. (2019) found a causal positive association between Lp(a) levels and LAS and, remarkably, an inverse association with SVS and Alzheimer disease (AD). Using individual-level data from the UK Biobank, Larsson et al. (2020b) confirmed a positive association with any ischemic stroke but could not replicate the protective association with AD. The latter result, however, could have been due to lack of statistical power, as the investigators did find a protective association with parental AD or dementia.

### Type 2 DM

Extensive data point to a deleterious effect of diabetes on ischemic stroke, but there is no such evidence for hemorrhagic stroke. Larsson et al. (2017) used MR analyses to confirm a causal relationship between type 2 diabetes mellitus (T2DM) and risk of ischemic stroke, especially LAS, but found null associations between other metabolic markers such as glucose, insulin levels, and body mass index (BMI). Gan et al. (2019) replicated this causal association between T2DM and ischemic stroke in the CKB, and Liu et al. (2018) found an association between T2DM and lacunar stroke. Similarly, using a combined exposure comprising phenotypes related to insulin resistance (fasting insulin adjusted for BMI, HLD-C and triglycerides, and insulin sensitivity), Chen et al. (2020) found a causal relationship between insulin resistance and ischemic stroke, particularly small vessel stroke. On the other hand, Yeung et al. (2018) did not find an association between glycated hemoglobin (HbA1c) and ischemic stroke in an MR study within the UK Biobank. More recently, Georgakis et al. (2021) investigated the effects of T2DM, hyperglycemia, insulin resistance, and β-cell dysfunction on the risk of stroke and related traits, finding that type 2 diabetes and higher HbA1c levels are associated with higher risk stroke, and particularly of LAS and SVS, with similar associations found for insulin resistance and β-cell dysfunction. β-Cell dysfunction was also associated with the risk of ICH. Furthermore, a study focused on neuroimaging markers of cerebrovascular disease found that type 2 diabetes was also positively associated with fractional anisotropy (a measure of white matter integrity) and inversely associated with gray matter volume and total brain volume, with similar inverse associations seen for HbA1c and β-cell dysfunction.

### Smoking

A large body of literature indicate that smoking is associated with both ischemic stroke and aneurysmal subarachnoid hemorrhage (Peters et al., 2013). Qian et al. (2019) reported a causal association between smoking and any ischemic stroke and LAS. In other MR studies, Larsson et al. (2019a, 2020c) confirmed the association between smoking initiation and the risk of any ischemic stroke, LAS and SVS, but did not find an association with cardioembolic stroke (CES) or ICH. In a one-sample MR study using the UK Biobank, Acosta et al. (2021) confirmed an association between genetic propensity to smoke and risk of nontraumatic subarachnoid hemorrhage. Similarly, in a large GWAS of intracranial aneurysms and subarachnoid hemorrhage, smoking was shown to be a powerful risk factor for this disease (Bakker et al., 2020).

### Obesity

Observational evidence suggests that obesity is a risk factor for stroke. However, MR studies have yielded heterogeneous results. One study indicated that while both general and central adiposity had causal effects on coronary heart disease, only central adiposity appeared to be associated with ischemic stroke (Dale et al., 2017). Further evidence supporting this hypothesis was provided by a more recent MR study by Marini et al. (2020) which found that higher waist-to-hip ratio, but not higher BMI, was causally associated with all-cause ischemic stroke, LAS, SVS, non-lobar ICH, and WMH volume.

### Lack of Physical Activity

Physical activity, especially moderate-to-vigorous physical activity, has been associated with lower risk of stroke in several observational studies. To date, only one MR has investigated this relationship, finding null results for this association (Zhuang et al., 2020). Therefore, further research is warranted in order to fully understand these conflicting results.

### Atrial Fibrillation

Atrial fibrillation (AF) is considered a major risk factor of cardioembolic ischemic stroke. Remarkably, patients with stroke are also at a higher risk of developing AF (Sposato et al., 2015, 2018). Only one MR study has looked at this relationship and, as expected given the overwhelming amount of observational evidence, found causal evidence to support this link using bidirectional MR (Hou et al., 2020).

## Nontraditional Risk Factors

A number of studies have explored nontraditional risk factors that had mixed or inconclusive evidence when evaluated in nongenetic, observational studies. Prominent among these are studies that focused on inflammation, coagulation factors, sleep health, and nutrition. MR studies have evaluated several of these factors in search of causal associations (Table 3).

**Table 3:** Summary of MR studies looking at nontraditional risk factors for stroke.

References	Exposure	Outcomes	Findings
Larsson et al., 2020a	Alcohol	CVDs	Causal relationship between higher alcohol consumption and increased risk of stroke and peripheral artery disease.
Yuan et al., 2020c	PUFA	IS	High-level plasma ALA was protective for IS, but AA was the opposite. LA, EPA, DHA, and DPA had no effects on IS.
Au Yeung and Schooling, 2020	Urinary sodium	CVDs	Higher log-transformed urinary sodium was associated with higher risk of stroke.
Larsson et al., 2019b	Serum magnesium and calcium	IS	Genetically higher serum magnesium concentrations were associated with a reduced risk of cardioembolic stroke but found no significant association of genetically higher serum calcium concentrations with any IS subtype.
Wang et al., 2021	Tea	IS	Genetically predicted an extra daily cup of tea consumption was causally associated with a reduced risk of small vessel stroke.
Qian et al., 2020	Coffee	IS and ICH	Coffee consumption was not causally associated with risk of stroke or its subtypes.
Choi et al., 2020	Serum bilirubin levels	IS an ICH	Causal associations between serum bilirubin levels and decreased stroke risk.
Huang et al., 2019	25(OH)D	CVDs	No evidence to support that genetically increased 25(OH)D was associated with a lower risk of IS, ICH, and SAH.
Larsson et al., 2018b	25(OH)D	IS	No evidence that genetically determined higher S-25OHD concentrations were causally associated with any ischemic stroke subtype.
Larsson et al., 2018a	VitK_1_	IS	Genetic predisposition to higher circulating vitamin K_1_ levels was associated with an increased risk of large artery atherosclerotic stroke.
Schooling et al., 2018	Testosterone	CVDs	Genetically determined testosterone was associated with IS.
Zhang et al., 2020	CRP	IS	No clear support that genetically determined elevated CRP concentration was causally associated with the risk of IS.
Yuan et al., 2020b	Circulating ILs	CVDs	Positive association of IL-1 with cardioembolic stroke and suggestive inverse associations of IL-6 with any IS, cardioembolic stroke, and small vessel stroke, and of IL-16 with CAD.
Georgakis et al., 2020d	Genetic proxies for IL-6R-mediated downregulation of IL-6 signaling	IS and other CVDs	Genetically downregulated IL-6 signaling to be associated with lower risks of IS.
Yuan et al., 2020a	TNF	CVDs	Genetically predicted TNF levels were positively associated with coronary artery disease and IS.
Wang et al., 2020	GDF-15	CVDs	Causal relationship of circulating GDF-15 levels with the increased risk of cardioembolic stroke, atrial fibrillation, coronary artery disease and myocardial infarction, but not any IS, large-artery atherosclerotic stroke, small vessel stroke, heart failure, and nonischemic cardiomyopathy.
Song et al., 2020	TIM-1	Stroke	Causal effect of TIM-1 on stroke.
Georgakis et al., 2019	Cytokines	Stroke	Genetic predisposition to elevated circulating levels of MCP-1 was associated with higher risk of stroke, in particular with large-artery stroke and cardioembolic stroke.
Lin et al., 2020	Inflammatory biomarkers	IS	No convincing evidence to support that inflammatory biomarkers like IL-1Ra, sIL-6R, and CRP were causally associated with the risk of IS or its subtypes.
Gill et al., 2018c	Iron	Stroke	MR evidence that higher iron status was associated with increased stroke risk and, in particular, CES.
Titova et al., 2020	Sleep duration	Stroke	No clear support that a genetically determined short or long sleep duration has influence on the risk of total stroke or stroke types.
Lu et al., 2020	Sleep duration	Stroke	Sleep duration was not causally associated with risk of stroke and its subtypes.
Cai et al., 2020	Sleep traits	IS	Potential causal role of short sleep duration and insomnia symptoms in LAS.
Larsson and Markus, 2019	Insomnia	CAD and stroke	Causal link between insomnia and ischemic stroke and its subtypes.
Marouli et al., 2020	Thyroid hormones	Stroke or CAD	A 1-SD increase in TSH was associated with a 5% decrease in the risk of stroke.
Cai et al., 2019	MDD	SVS	Possible causal effect of MDD on increased risk of SVS.
Gill et al., 2019b	Depression	IS and functional outcome after IS	No evidence of genetically determined risk of depression affecting IS risk but consistent MR evidence suggestive of a possible effect on functional outcome after IS.
Gill et al., 2018b	Platelet count	CVDs	Higher genetically determined platelet count was causally associated with higher risk of IS.
Gill et al., 2018a	FXI	IS, ICH, MI	Causal effect of higher, genetically determined FXI levels on risk of any IS.
Harshfield et al., 2020	Hematological traits	IS and its subtypes	Several factors on the intrinsic clotting pathway were significantly associated with CES and LAS, but not with SVS. On the common pathway, increased gamma (γ’) fibrinogen was significantly associated with AIS/CES. Furthermore, elevated plateletcrit was significantly associated with AIS/CES, eosinophil percentage of white cells with LAS, and thrombin-activatable fibrinolysis inhibitor activation peptide antigen with AIS. Follow-up analysis in UK Biobank showed that among individuals with atrial fibrillation, those with genetically lower levels of factor XI are at reduced risk of AIS compared to those with normal levels of factor XI.
Jia et al., 2019	Gut microbiota dependent metabolites (betaine, carnitine, choline, and trimethylamine N-oxide)	Stroke	No association.

*CVDs, cardiovascular disorders; ICH, intracerebral hemorrhage; IS, ischemic stroke; PUFA, polyunsaturated fatty acids; AA, arachidonic acid; LA, linoleic acid; EPA, eicosapentaenoic acid; DHA, docosahexaenoic acid; DPA, docosapentaenoic acid; SAH, subarachnoid hemorrhage; FAs, circulating fatty acids; CRP, C-reactive protein; ILs, interleukins; IL-1, interleukin 1; IL-6, interleukin 6; IL-16, interleukin 16; TNF, tumor necrosis factor; GDF-15, growth differentiation factor 15; TIM-1, T-cell immunoglobulin and mucin-1; MCP-1, monocyte chemoattractant protein-1; LAS, large artery stroke; CAD, coronary artery disease; TSH, thyroid stimulating hormone; MDD, major depressive disorder; SVS, small vessel stroke; MR, Mendelian randomization; FXI, factor XI; MI, myocardial infarction; CES, cardioembolic stroke; and AIS, arterial ischemic stroke*.

### Inflammatory Biomarkers

Observational studies have demonstrated that patients with underlying inflammation have a higher risk of stroke (Anrather and Iadecola, 2016), although this association could be the result of confounding. Therefore, genetically determined inflammatory biomarkers have been targeted by many studies investigating the risk of stroke. Zhang et al. (2020) examined whether genetically raised plasma C-reactive protein (CRP) concentration levels were associated with ischemic stroke without finding a significant association. In line with these findings, Lin et al. (2020) evaluated numerous inflammatory biomarkers and found no association between genetically elevated levels of these biomarkers and ischemic stroke. Yuan et al. (2020b) analyzed genetically determined circulating interleukins in relation to coronary artery disease (CAD), atrial fibrillation, and ischemic stroke, and its subtypes. There was a suggestive (*p* < 0.05 but not statistically significant after correction for multiple testing) positive association between interleukin-1 receptor antagonist and cardioembolic stroke and a suggestive inverse association between interleukin-6 and ischemic stroke, CES, and SVS, and of interleukin-16 with CAD. Georgakis et al. (2020d) reported the results of an MR study focused on interleukin-6 signaling effects on ischemic stroke and other cardiovascular outcomes and demonstrated that genetically downregulated interleukin-6 signaling was associated with lower risk of ischemic stroke. The same group published a study investigating genetically determined levels of circulating cytokines and risk of stroke (Georgakis et al., 2019). They showed that genetic predisposition to elevated circulating levels of monocyte chemoattractant protein-1 was associated with higher risk of stroke, in particular with LAS and CES. Another study by Yuan et al. (2020a) investigated causal associations of increased tumor necrosis factor levels and several highly prevalent CVDs. Genetically elevated tumor necrosis factor levels were positively associated with both CAD and ischemic stroke. In addition, Wang et al. (2020) investigated the impact of growth differentiation factor 15 on the risk of CVDs using an MR approach. They found evidence for a relationship between circulating of growth differentiation factor 15 levels and increased risk of CES, atrial fibrillation, CAD, and myocardial infarction. Finally, Song et al. (2020) published results of their study investigating association of genetically determined T-cell immunoglobulin and mucin domain 1 with incidence of stroke, which found a causal effect of TIM-1 on any stroke and ischemic stroke.

### Hematological Traits

The role of hematological traits and pathways in the occurrence of stroke has also been extensively evaluated using MR. Gill et al. (2018b) investigated genetically determined platelet count and risk of different CVDs, finding that higher genetically determined platelet count is associated with higher risk of ischemic stroke. The same group released results of an MR study focused on genetically determined factor XI levels that found causal evidence supporting factor XI as a possible target to reduce the risk of cardioembolic stroke (Gill et al., 2018a). Harshfield et al. (2020) reported a study that, rather than focusing on a single target, evaluated several different hematological traits in connection to risk of ischemic stroke and its subtypes. Several factors in the intrinsic coagulation pathway were significantly associated with CES and LAS but not with SVS. Specifically, gamma fibrinogen, a component of the common pathway, was associated with CES, plateletcrit was associated with CES, eosinophil percentage of white cells was associated with LAS, and thrombin-activatable fibrinolysis inhibitor activation peptide antigen was associated with any ischemic stroke. Follow-up analyses in the UK Biobank showed that among individuals with atrial fibrillation, those with genetically lower versus normal levels of factor XI have a reduced risk of ischemic stroke. Finally, one group investigated circulating vitamin K_1_ levels in connection to cerebrovascular disease and found that genetic predisposition to higher circulating vitamin K₁ levels was associated with an increased risk of LAS (Larsson et al., 2018a).

### Nutritional Factors

A number of MR studies also investigated the role of nutritional factors in the occurrence of stroke, another research avenue extensively explored from an observational perspective. Larsson et al. (2020a) examined whether genetically determined predisposition to alcohol consumption had influence on risk of CVD. This study provided evidence for a causal relationship between higher alcohol consumption and increased risk of any stroke and peripheral artery disease.

Beyond alcohol, several other nutritional factors have been investigated. One research group examined the role of plasma phospholipid fatty acids in risk of 15 CVD-related phenotypes. Genetically higher plasma α-linolenic, linoleic, and oleic acid levels were inversely associated with LAS and venous thromboembolism, whereas arachidonic and stearic acid levels are positively associated with these two CVD-related outcomess (Yuan et al., 2019).

Iron metabolism, including anemia and polycythemia, have long been postulated to play a role in cerebrovascular disease. Gill et al. (2018c) published results of an MR study investigating this question, finding that higher iron levels were associated with increased risk of stroke and, in particular, CES.

Two groups reported on genetically predicted levels of vitamin D and the risk of stroke. Huang et al. investigated whether vitamin D played a role in risk and mortality of several vascular diseases by conducting an MR study that included both Asian and European participants (Huang et al., 2019). They found no evidence to support that genetically increased vitamin D was associated with a lower risk of ischemic stroke, ICH, SAH, and lipid levels in neither Chinese nor European. Similarly, Larsson et al. (2018b) reported the results of a study on genetically determined vitamin D concentrations and ischemic stroke and its subtypes that failed to find significant associations.

### Other Risk Factors

Among other investigated risk factors, it is worth mentioning two studies investigating endocrine changes. Schooling et al. (2018) analyzed genetic predictors of testosterone and their associations with different CVD phenotypes. They confirmed prior results from observational studies showing a significant association between genetically proxied testosterone and risk of ischemic stroke. Another study by Marouli et al. (2020) used MR analyses to analyze whether thyroid function affects the risk of stroke *via* atrial fibrillation, finding that a 1 SD increase in TSH was associated with a 5% decrease in the risk of ischemic stroke.

Finally, another four studies are also worth mentioning. Larsson et al. (2019b) examined serum magnesium and calcium levels in relation to ischemic stroke using MR. They found that genetically higher serum magnesium concentrations were associated with a reduced risk of CES but not with other stroke subtypes. Au Yeung and Schooling (2020) investigated the impact of urinary sodium on CVD. Higher genetically determined log-transformed urinary sodium was associated with higher risk of stroke. Choi et al. (2020) used MR tools to describe a causal association between serum bilirubin levels and decreased stroke risk. Lastly, Jia et al. (2019) investigated the role of gut microbiota-dependent metabolites and risk of several CVDs, finding no evidence of a causal link between these metabolites (betaine, carnitine, choline, and trimethylamine N-oxide) and the risk of stroke.

## Mendelian Randomization for Drug Target Validation and Drug Repurposing

Beyond hypothesis-driven MR studies, population genetics offers powerful tools to accelerate the discovery of novel biological pathways by agnostically evaluating several biological targets and/or reevaluating targets for drug repurposing (Nelson et al., 2015; Schmidt et al., 2020). This approach is significantly potentiated by the growing culture of open-access research and the increasing availability of high-throughput genomic and proteomic technologies.

Drug target MR studies use genetic variants that lie within or near the genes coding for these targets (the latter called cis-variants) as instruments, which either have an effect on the actual serum levels of the target protein or other endpoint, such as an intermediate biomarker, gene expression levels, or metabolite levels. This distinction is important because drug target MR aims to answer a different question than conventional MR. While conventional MR establishes causal relationships between biomarkers or traits and an outcome, drug target MR addresses whether modifications of a specific drug target or protein will have an effect on the outcome (Gill et al., 2021).

Drug target MR overcomes problems related to pleiotropy, which are especially relevant when looking at possible targets for intervention. In addition, by testing these drug targets phenome-wide, investigators can also pinpoint possible adverse effects. While drug target MR is a robust methodology, it also has limitations. While new high-throughput technologies have increased the amount of proteome-wide data available, publicly available summary statistics and proteomic studies with adequate sample sizes are still limited. This relative paucity of proteomic data leads to the utilization of cis-variants associated with a biomarker in the causal pathway, which could in turn lead to some problems. For example, circulating levels of a biomarker could not represent accurately its cellular concentration, which is often the value of interest, and this could limit the ability to detect causality. Additionally, the biomarker of interest could be relevant only in certain physiological or disease states, or during a critical period of time, which could also limit results. Lastly, not all proteins are druggable (i.e., able to be pharmacologically manipulated), which of course would defeat the purpose of doing drug target MR (Mokry et al., 2015).

Gill et al. (2019a) compared the results of drug target MR and clinical trials for three antihypertensive drug classes (angiotensin-converting-enzyme inhibitors, β-blockers, and calcium channel blockers) in coronary heart disease and stroke, finding comparable estimates. Along these lines, Chong et al. (2019) conducted a proteome-wide MR study to investigate potential therapeutic targets in ischemic stroke. The authors analyzed 653 circulating proteins as possible causal factors for the three main subtypes of ischemic strokes (LAS, SVS, and CES) and hemorrhagic stroke. In their analyses, they found eight biomarker–stroke associations encompassing seven unique targets. Of these biomarkers, five had already been associated with CVDs, including the coagulation factor 11, Lp(a), ABO, CD40 (a member of the tumor necrosis factor superfamily), and MMP12 (a member of the matrix metalloproteinase family, implicated in vascular remodeling). Novel biomarkers included SCARA5, a protein with a role in iron homeostasis, and TNFS12, a pleiotropic tumor necrosis factor-like cytokine linked to atrial fibrillation, a potential mediating mechanism.

Drug repurposing is another potentially useful approach, as previously approved drugs can be more easily brought to clinical practice if a beneficial effect is found. There are no clear examples of MR studies specifically focused on drug repurposing for stroke. However, we bring the reader’s attention to two such studies that explored the potential of repurposing medications to treat Alzheimer’s disease, unfortunately with null results (Walker et al., 2020; Wu et al., 2021). These studies provide an appropriate example and analytical framework for future studies applying this approach to stroke and cerebrovascular diseases.

## Conclusion

Stroke constitutes an increasingly prevalent condition worldwide and remains one of the leading causes of death and disability. MR has proved to be a powerful methodology to confirm or refute associations described by observational studies and identify novel therapeutic targets for stroke. The ability of MR studies to add valuable scientific evidence to the field of cerebrovascular disease research will be greatly increased by the utilization of novel tools, including proteome-wide and drug target validation analyses.

## Author Contributions

JA, NS, and GF reviewed the literature, drafted the manuscript, and revised the manuscript for intellectual content. All authors contributed to the article and approved the submitted version.

## Conflict of Interest

The authors declare that the research was conducted in the absence of any commercial or financial relationships that could be construed as a potential conflict of interest.

## Publisher’s Note

All claims expressed in this article are solely those of the authors and do not necessarily represent those of their affiliated organizations, or those of the publisher, the editors and the reviewers. Any product that may be evaluated in this article, or claim that may be made by its manufacturer, is not guaranteed or endorsed by the publisher.
